# Internal Flames: Metal(loid) Exposure Linked to Alteration of the Lipid Profile in Czech Male Firefighters (CELSPAC-FIREexpo Study)

**DOI:** 10.1021/acs.estlett.4c00272

**Published:** 2024-06-12

**Authors:** Nina Pálešová, Katarína Řiháčková, Jan Kuta, Aleš Pindur, Ludmila Šebejová, Pavel Čupr

**Affiliations:** † RECETOX, Faculty of Science, Masaryk University, Kamenice 753/5, 625 00 Brno, Czech Republic; ‡ Faculty of Sports Studies, Masaryk University, Kamenice 753/5, 625 00 Brno, Czech Republic; § Training Centre of Fire Rescue Service, Fire Rescue Service of the Czech Republic, Ministry of the Interior, Trnkova 85, 628 00 Brno, Czech Republic

**Keywords:** firefighters, occupational exposure, metals, cholesterol, cardiovascular disease, mixture analysis

## Abstract

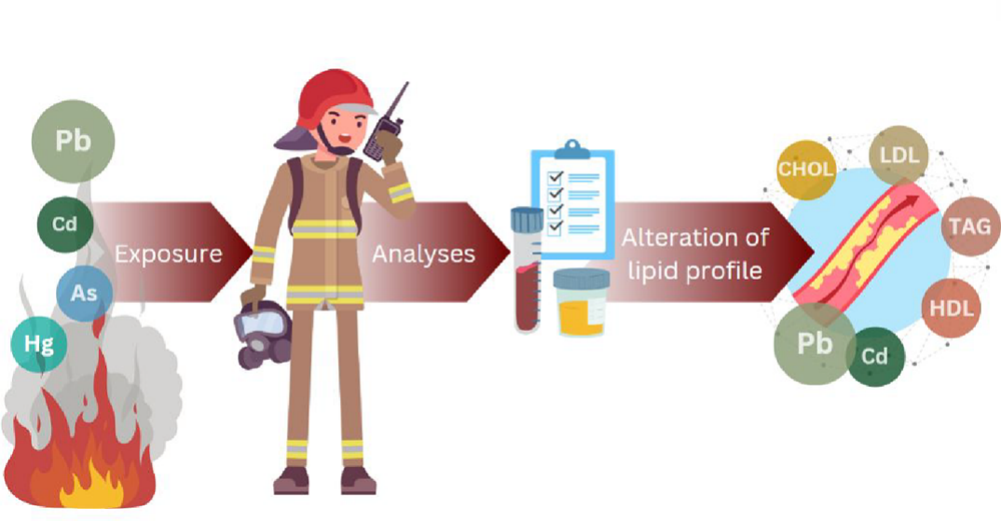

Increased wildfire activity increases the demands on fire rescue services and firefighters’ contact with harmful chemicals. This study aimed to determine firefighters’ exposure to toxic metal(loid)s and its association with the lipid profile. CELSPAC-FIREexpo study participants (including 110 firefighters) provided urine and blood samples to quantify urinary levels of metal(loid)s (arsenic, cadmium (Cd), mercury, and lead (Pb)), and serum lipid biomarkers (cholesterol (CHOL), low-density lipoprotein cholesterol (LDL), high-density lipoprotein cholesterol (HDL), and triglycerides (TG)). The associations were investigated by using multiple linear regression and Bayesian weighted quantile sum (BWQS) regression. Higher levels of Pb were observed in firefighters. Pb was positively associated with CHOL and TG. Cd was negatively associated with HDL. In the BWQS model, the mixture of metal(loid)s was associated positively with CHOL (β = 14.75, 95% CrI = 2.45–29.08), LDL (β = 15.14, 95% CrI = 3.39–29.35), and TG (β = 14.79, 95% CrI = 0.73–30.42), while negatively with HDL (β = −14.96, 95% CrI = −25.78 to −1.8). Pb emerged as a key component in a metal(loid) mixture. The results suggest that higher exposure to lead and the mixture of metal(loid)s is associated with the alteration of the lipid profile, which can result in an unfavorable cardiometabolic profile, especially in occupationally exposed firefighters.

## 1 Introduction

Climate data and projections for Czechia suggest a tendency toward warmer average temperatures and reduced warm-season precipitation, which contributes to greater wildfire activity.^1,2^ Increased incidence of wildland fires over the last 10 years showed that Czechia, although placed in Central Europe, is not immune to this climate change fueled natural hazard.^3^ Czechia has 11.7% of its land area in the wildland–urban interface (WUI), the intersection between wildland vegetation and urban housing. This exceeds the European average (7.4%), raising concerns about increasing wildfire hazards, including emissions from burning biomass and man-made materials.^4^ Toxic metal(loid)s are present at low concentrations in the environment (naturally or due to the contamination, e.g., leaded gasoline^5,6^), while at higher concentrations in man-made materials and products (e.g., electric vehicles,^7^ photovoltaic systems,^8^ lead paints, arsenic wood preservatives^9^). Even trace amounts of metal(loid)s in particular matter (PM) produced by burning of materials can lead to sizable and toxicologically relevant emissions.^10,11^ For example, during the 2018 Camp Fire in California, atmospheric lead levels at a monitoring station 200 km away increased 40-fold.^12^ In Greece, wildfire ash contained heavy metals exceeding residential soil screening levels, posing potential health risks.^9^

Firefighters are on the front line of hazardous chemical exposures, and despite their use of personal protective equipment (PPE) and self-contained breathing apparatus (SCBA, not used during wildfires), they are exposed via multiple routes including inhalation and dermal contact.^13^ Higher incidence rates of cardiovascular disease (CVD) have been recorded in firefighters compared to the general population.^14^ Besides lifestyle factors, such as stress, diet, long hours, and shift work, occupational exposure to harmful chemicals during both real fire suppression activities and training fires might contribute to higher incidence. Toxic metals and metalloids such as arsenic (As), cadmium (Cd), mercury (Hg), and lead (Pb) are of significant concern owing to their release during combustion^12^ (from contaminated soil and building materials (e.g., Pb content in paints, solder, and old pipes; Hg in fluorescent lamps; varying amounts of Cd, Pb, and other metals in rechargeable batteries; and As content in wood preservatives))^9^ and potential to cause adverse health effects, including hyperlipidemia, a crucial risk factor for CVD.^15−18^

Several studies focused on both internal and external exposure of firefighters to metals, comprehensively indicating their significant occupational exposure.^19−21^ However, only a few studies linked the exposure with CVD-related health outcomes. Blood Cd levels were significantly associated with increased risk of metabolic syndrome and hypertension among Korean firefighters.^22,23^ Furthermore, potential toxicological interactions between individual metals as well as with other fire emissions (such as polycyclic aromatic hydrocarbons (PAHs)) were reported.^20,24,25^ However, available studies focused on individual metals, and exposure to metal mixtures has not yet been investigated among firefighters.

Approximately 74,300 firefighters (professional and voluntary) in Czechia can potentially be involved in the first responses to incidents (data to 2020),^26^ which represents almost 0.7% of the Czech population. Despite the exacerbation of fire hazards over the past 15 years by the escalating climate crisis and grow of WUI, and evidence of metal(loid) toxic effects,^15−18^ no human biomonitoring (HBM) study of metal(loid) exposure among firefighters has been carried out in Czechia nor anywhere in Central or Eastern Europe to date. Drawing conclusions from research conducted in different regions may introduce uncertainty due to variations in occupation and lifestyle factors and differing legislations and policies. Therefore, this study aimed to determine internal levels of As, Cd, Hg, and Pb in firefighters’ urine and their associations with firefighting activities; investigate the relationship between the metal(loid)s (individually as well as in complex mixture) and hyperlipidemia; and propose science-based recommendations to prevent and/or mitigate the risks arising from firefighters’ occupational exposure to metal(loid)s.

## 2 Methods and Materials

### 2.1 Study Population and Study Design

The study population is described in detail previously.^27^ In brief, between 2019 and 2020, the CELSPAC-FIREexpo study included 166 participants to assess firefighters’ chemical exposure during firefighting and training. All participants were physically active men from Czechia, aged 18–35, and nonsmokers. Two participants withdrew during the study. Participants (*n* = 164) were categorized into three subcohorts according to their relationship with firefighting: newly recruited firefighters after 15-week professional training program prior to becoming active firefighters (“NEW FF”; *n* = 58), professional firefighters actively participating in incidents (“PROF”; *n* = 52), and a control group of nonfirefighters (“CTRL”; *n* = 54). Participants from NEW FF subcohort were exposed to fire during firefighting training in an indoor environment 4 weeks prior the sampling. All participants answered questionnaires and provided morning void urine for the analyses of metal(loid)s and a fasting blood sample for the analyses of serum lipid biomarkers. The study was approved by the ELSPAC Ethics Committee in 2019, and all participants gave written informed consent. Information regarding the transportation and storage of samples and the questionnaires is available in the Supporting Information (sample collection and storage; Table S1).

### 2.2 Assessment of Exposure and Serum Lipid Biomarkers

The concentration of three metals (Cd, Hg, and Pb) and one metalloid (As) in urine samples was determined by inductively coupled plasma mass spectrometry (Agilent 8900 ICP-MS/MS, Agilent Technologies).^28^ Information regarding the details of the method and QA/QC is available in the Supporting Information (section Quality control and quality assurance).

Urine creatinine levels and specific gravity (SG) were determined for the adjustment of urinary metal(loid) levels, using LC–MS/MS^29^ and hand-held PAL-10 S refractometer (Altago, Japan), respectively. Formulas used for the adjustments are presented in the Supporting Information (Table S2).

The levels of total cholesterol (CHOL, mmol/L), low-density lipoprotein (LDL, mmol/L), high-density lipoprotein (HDL, mmol/L), and triglycerides (TG, mmol/L) in serum were considered markers of the lipidic profile and were measured spectrophotometrically with an Alinity c instrument (Abbott, Illinois, U.S.A) (Supporting Information section Quality control and quality assurance).

### 2.3 Statistical Analyses

SG-adjusted urine metal(loid) levels were used for calculation of descriptive statistics and further statistical analyses.^30^ SG-adjusted urinary metal(loid)s as well as serum lipid biomarkers were log 2 transformed to address skewness and improve the normality of the distribution. Spearman’s correlation coefficients were calculated to evaluate pairwise correlations between individual metal(loid)s, demographic characteristics of the study population, and lipid biomarkers. Statistical differences between the subcohorts were investigated by ANOVA/Kruskal–Wallis ANOVA with Tukey/Wilcox post hoc tests and χ^2^ test with post hoc tests. After the standardization of the data for the interquartile range (to minimize the influence of outliers), linear regression models were used to investigate the associations of urinary metal(loid)s with study population characteristics, such as age (in years), body mass index (BMI) (in kg/m^2^), former smoking (yes/no), study subcohort (CTRL/NEW/PROF), length of FF career (in years), and contact with large fire in the last six months (never/one time/two or more times). Using individual multiple linear regression (MLR), associations between each urinary metal(loid) and each lipid biomarker were examined. A set of covariates was identified based on *a priori* knowledge, a directed acyclic graph (DAG) approach (Figure S1),^31^ and the results from linear regression models: age, BMI, previous smoking, length of firefighting career, and study subcohorts. Second, associations between metal(loid) mixture and lipid biomarkers were assessed using Bayesian weighted quantile sum (BWQS) regression.^32−34^ Results from MLR and BWQS are expressed as the relative change in the median of lipid biomarkers for a doubled concentration of metal(loid)s in urine.

Several sensitivity analyses were performed. First, to explore the potential effect of extreme values and nonlinear associations, urinary metal(loid) levels were categorized into quartiles, and then multiple linear regression was performed. Second, MLR and BWQS models were performed on a reduced data set (*n* = 106), excluding the NEW FF subcohort due to the potentially significant effect of intense physical activity during training program. Third, to assess complex relationships of exposure to fire emissions, 6 urinary metabolites of polycyclic aromatic hydrocarbons (PAHs) were additionally included in the mixture BWQS model. Naturally, PAHs and metals occur together in fire emissions and data of urinary levels of hydroxylated metabolites of PAHs (OH-PAHs) are available for this cohort and were previously described in detail.^27,35^ All statistical analyses were performed using Rstudio version 4.2.3.^36^

## 3 Results and Discussion

### 3.1 Study Population Characteristics and Urinary Metal(loid) Levels

Table 1 presents the characteristics of the study population. The participants were 26.4 years old on average, with PROF being the oldest, which corresponds to the length of the firefighting career. Firefighters (both PROF and NEW FF) had higher BMI compared to CTRL. The highest rate of former smoking was reported among the PROF. PROF had higher levels of CHOL and LDL compared to other subcohorts. They also had a higher proportion of individuals above physiological limits for the LDL.

**Table 1:** Characteristics of the Study Population^a^

		**Overall study population**	**NEW FF**	**PROF**	**CTRL**
		*n* = 164	*n* = 58	*n* = 52	*n* = 54
**Characteristics**		**Mean ± SD**	**Mean ± SD**	**Mean ± SD**	**Mean ± SD**
**Age (years)**		26.4 ± 4.3	25.0 ± 3.6	28.4 ± 3.6	26.0 ± 4.9
**BMI**		25.82 ± 2.70	26.31 ± 2.83	26.14 ± 2.35	24.99 ± 2.72
**Former smoking (%)**					
**yes**		21 (13%)	7 (12%)	10 (19%)	4 (7.4%)
**no**		143 (87%)	51 (88%)	42 (81%)	50 (92.6%)
**Length of FF career (years)**		1.76 ± 2.77	0.88 ± 0.67	4.58 ± 3.43	n.a.
**Contact with large fire in the last 6 months (%)**	Never	52%	47%	19%	91%
	Once	20%	33%	21%	0.04%
	Twice and more	46%	21%	60%	0.06%

**Biomarkers**	**Out of normal range**		**Median (25th; 75th percentile)**			
			**Overall study population**	**NEW FF**	PROF	CTRL
**CHOL** (mmol/L)			4.50	4.20	4.90	4.45
			(4.00; 5.20)	(3.99; 4.80)	(4.38; 5.43)	(3.70; 5.18)
	>5.00		29.3%	12.1%	48.1% ×	29.6%
**LDL** (mmol/L)			2.80	2.65	3.15 *	2.75
			(2.40; 3.30)	(2.35; 3.10)	(2.73; 3.67)	(2.23; 3.30)
	>3.00		38.4%	29.3%	55.8% *	31.5%
**HDL** (mmol/L)			1.35	1.40	1.35	1.30
			(1.20; 1.50)	(1.10; 1.40)	(1.20; 1.50)	(1.13; 1.50)
	<1.00		2.5%	1.7%	4.2%	1.9%
**TAG** (mmol/L)			1.06	1.02	1.15	1.02
			(0.81; 1.47)	(0.82; 1.49)	(0.87; 1.59)	(0.78; 1.17)
	>1.70		18.3%	15.5%	23.1%	16.7%

a *x*, statistically different from new firefighters after training; *, statistically different from both new firefighters after training and controls. Measurement of HDL was available only for 160 participants.

**Figure 1:**
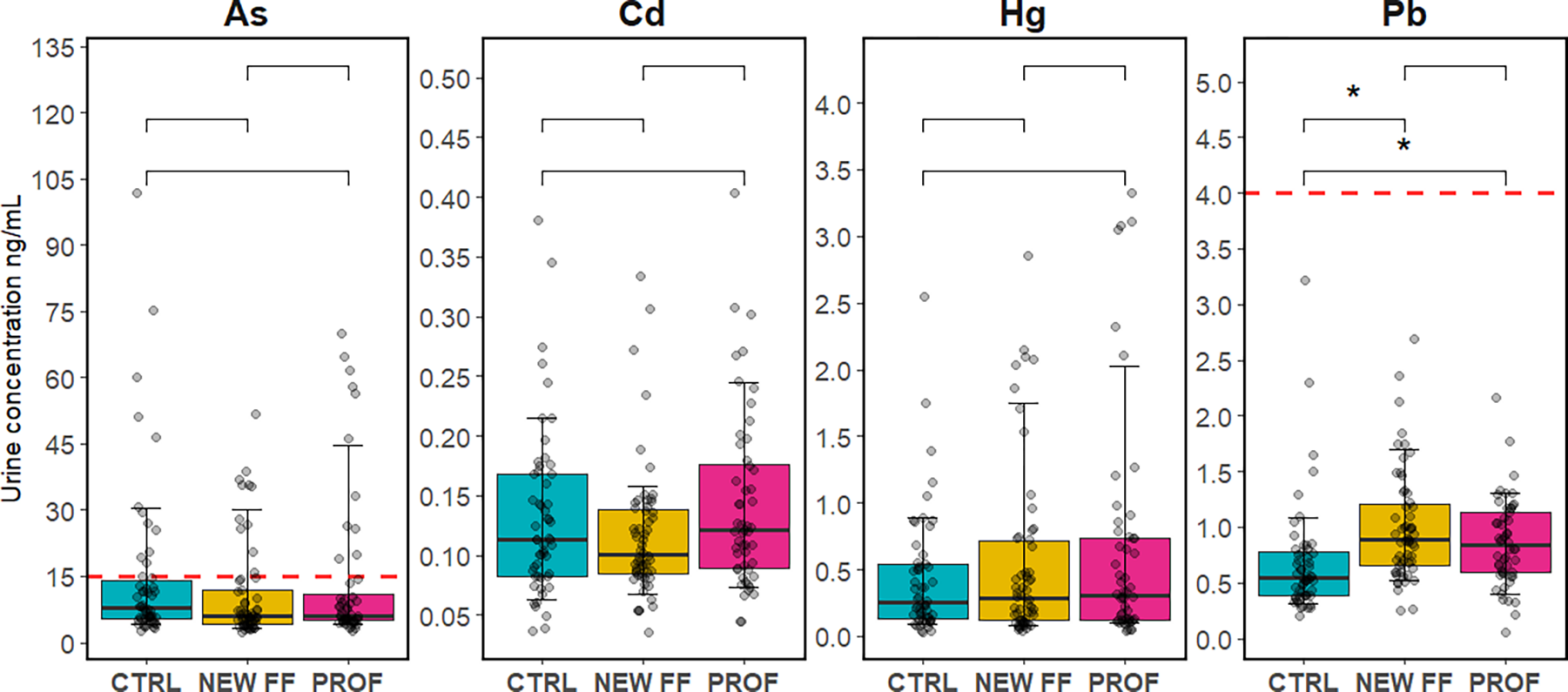
SG-adjusted levels of urinary metals (ng/mL) in 3 study subcohorts - PROF, NEW FF, and CTRL. “*” indicates a statistically significant difference (*p* < 0.001). Red dashed lines represent the human biomonitoring reference value (RV95) statistically derived from the studies conducted on general populations (RV95 (As) = 15 ng/mL;^37^ RV(95) Pb = 4 ng/mL^38^).

As, Cd, Hg, and Pb were detected in all analyzed samples of urine, with As having the highest median (6.3 ng/mL), followed by Pb (0.74 ng/mL), Cd (0.11 ng/mL), and Hg with the lowest median (0.29 ng/mL) (Tables S3 and S4). When participants were stratified into study subcohorts, firefighters had 1.5–1.6 times higher levels of urinary Pb compared to CTRL (*p* < 0.001), while As, Cd, and Hg were not statistically different across the subcohorts (Figure 1). As was positively correlated (*p* < 0.05) with Cd (0.153) and Hg (0.198), while Pb was not correlated with any other metals. This implies the similarity of As exposure pathways to those of Cd and Hg and heterogeneity for Pb. Pb was positively correlated (*p* < 0.05) with the length of FF career (0.278) and frequency of contact with fire (0.180), suggesting FF occupation might be a significant exposure source for Pb (Figure S2). Regression analysis revealed significant positive association between urinary As and BMI (β = 17.04%; CI = 5.73–29.57%) and negative association between Pb and BMI (β = −10.43%; CI = −18.97 to −0.98%). Urinary Cd, Hg, and Pb were positively associated with age (Table S5). Moreover, significant positive association between urinary Pb and firefighting occupation (β = 31.43%, CI = 6.97–61.48% for PROF, and β = 54.92%, CI = 26.79–89.31% for NEW FF), length of FF career (β = 9.02%, CI = 1.55–17.04%) and a single contact with large fire in the last 6 months (β = 30.24%, CI = 3.68–63.61%) were observed (Table S5), which suggest that firefighting activities notably contribute to firefighters’ Pb exposure.

Median urinary As measured in Turkish firefighters was 15.65 ng/mL,^39^ which is substantially higher compared to the median in PROF in our study (6.1 ng/mL). A higher median of As was observed also in Californian firefighters (10.4 ng/mL).^21,40^ Observed differences might be caused by the natural occurrence of As in the environment and regionally different procedures applied during firefighting and decontamination of the gear. Medians of urinary Cd and Hg observed in Californian firefighters in the FOX study were 0.138 ng/g creatinine and 0.45 ng/mL, respectively,^40^ which is comparable with our study (Tables S3 and S4), suggesting a similar chemical burden of both Czech and Californian firefighters. No samples exceeded reference values (RV95, statistically derived from biomonitoring in general population) for Cd and Hg (derived from Czech population),^41^ while 20.7% values exceeded RV95 for As (derived from German population) (Figure 1).^37,42^ Similar As levels have been reported also in other European populations.^37^ Despite the specific occupational cohort included in this study, our results align with the general European population and suggest a decrease in Hg and Cd exposure, which is in line with decreased emissions across Europe.^37,41,43^

This is the first study to measure urinary Pb in firefighters. FOX study detected Pb in 100% of blood samples of Californian firefighters (median 9.5 ng/mL) and their results were lower compared to the general population in the USA (median 14.2 ng/mL, from National Health and Nutrition Examination Survey (NHANES)).^21^ In our study, significantly higher levels of Pb were observed in firefighters compared to those in the control population. Such inconsistency might arise from Pb toxicokinetics and different sample matrices. In blood, 99% of Pb is in erythrocytes and its excretion occurs through feces and urine. It is assumed that urine Pb mirrors the plasma’s filtratable fraction, which at constant exposure is in equilibrium with Pb in erythrocytes. However, when exposure changes, urinary Pb will change more rapidly, hence mirroring more recent exposures compared to blood Pb.^44^ No sample from our study exceeded the RV95 set for urinary Pb from the Belgian general population recruited in 2010/2011 (Figure 1),^38^ which corresponds with the substantial decrease of Pb emissions in Europe.^43^ However, our study indicates that despite effective policy measures, certain population groups remain disproportionately exposed, increasing their vulnerability.

Wildfires account for approximately 12% of all fire occurrences in Czechia, and the incidence rate is expected to increase in the future.^1,3^ In general, Pb in wildfire emissions is mostly associated with PM produced during specific circumstances aligned with structural burning (e.g., houses and vehicles), which is the case of fires in WUI. Moreover, wildfire burning can remobilize the Pb deposited before the phase-out of leaded gasoline.^12^ Firefighters can be heavily exposed to Pb via inhalation (SCBAs are not used during wildfires) and dermal route during fire suppression and overhaul phase.^13,21,45,46^ Additional exposure occurs dermally during postevent contact with contaminated surfaces (e.g., fire trucks, PPE, and other gear). Keir et al. (2020) reported a 9.1-fold and 3.5-fold increase of Pb on skin and PPE after fire suppression, respectively, and highlighted dermal exposure, postevent respiratory exposure (without SCBA), and dermal contact with contaminated gear as key components of firefighters’ Pb exposure.^13^

### 3.2 Associations between Metal(loid)s and Lipid Profile Biomarkers

**Figure 2:**
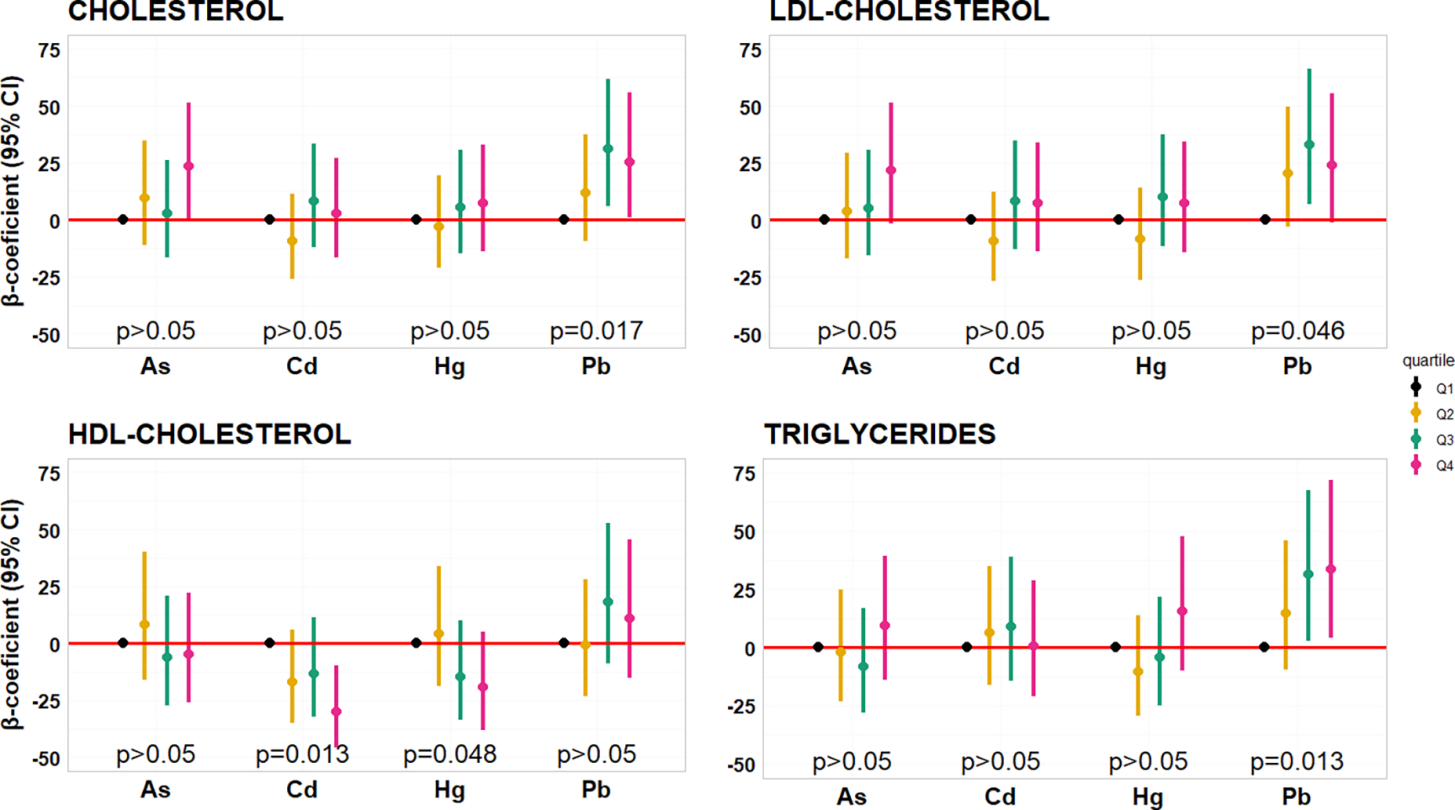
Associations between urinary metal(loid)s categorized into quartiles (Q1–Q4) and serum lipids (mmol/L): adjusted β-coefficients, 95% confidence intervals, and *p*-values for trend.

Urinary Pb levels exhibited positive associations with CHOL, HDL, and TG in the adjusted MLR models. Conversely, Cd was negatively associated with HDL (Table 2). Quartile categorization of metals yielded similar association patterns (Figure 2, Table S6). Although slight alterations in associations were noted upon exclusion of NEW FF from the data set, the directionality of β-coefficients remained consistent, and significant associations of Pb with CHOL and TG persisted (Table S7). Considering the metals collectively in the mixture by BWQS revealed a significant association with all lipid biomarkers: positive with CHOL, LDL, and TG, and negative with HDL (Table 2). Pb emerged as the key component of the mixture, except for association with HDL, where Cd predominated (Table S8). Excluding NEW FF data minimally impacted most of the BWQS model results, with only the association with HDL becoming nonsignificant (Table S7). Upon inclusion of six OH-PAHs in the model, significant associations remained only with CHOL and LDL (Tables S8 and S9). Observed differences may stem from the variability of toxicity mechanisms and/or interactions (both synergistic and antagonistic) caused by common signaling pathways of metals and PAHs.^25,47,48^

**Table 2:** β-Coefficients for the Associations between Urinary Metal(loid)s (ng/mL) Considered Individually (MLR Model, with 95% Confidence Intervals (CI)) as Well as in Mixtures (BWQS Model, with 95% Credibility Interval (CrI)) and Serum Lipids^a^

	**CHOL**	**LDL**	**HDL**	**TG**
	β (95% CI/CrI)	β (95% CI/CrI)	β (95% CI/CrI)	β (95% CI/CrI)
**As**	5.00 (−3.54, 14.29)	4.77 (−4.15, 14.51)	–3.76 (−13.32, 6.87)	4.03 (−5.69, 14.75)
**Cd**	5.16 (−3.9, 15.09)	7.78 (−1.9, 18.42)	**–15**.**03 (−23**.**65**, **–5**.**42)**	6.01 (−4.47, 17.64)
**Hg**	5.99 (−6.21, 19.78)	6.73 (−6.1, 21.32)	–13.13 (−25.18, 0.87)	7.67 (−6.5, 23.99)
**Pb**	**11**.**5 (1**.**53**, **22**.**45)**	8.9 (−1.35, 20.23)	**12**.**9 (0**.**72**, **26**.**56)**	**14**.**66 (2**.**94**, **27**.**71)**
**BWQS**	**14**.**75 (2**.**45**, **29**.**08)**	**15**.**14 (3**.**39**, **29**.**35)**	**–14**.**96 (−25**.**78**, **–1**.**8)**	**14**.**79 (0**.**73**, **30**.**42)**

a Models were adjusted for age, BMI, former smoking, length of firefighting career, and subcohort (CTRL/PROF/NEW FF). Results are expressed as percent change in lipid biomarker per doubling urinary metal(loid) concentration. **Bold** refers to statistical significance (*p* < 0.05).

The observed positive associations between Pb and CHOL, LDL, and TG and negative associations between Cd and HDL are consistent with the findings of previous studies of the general population, which reported relationships between metal exposure and an impaired lipidic profile and increased the incidence of metabolic syndrome (MetS), a cluster of interrelated metabolic disorders including dyslipidemia, abdominal obesity, high blood pressure, and hyperglycemia.^16,18,49−51^ However, there are only a few studies focused on firefighters’ metal exposure and its associations with the lipid profile. Recent studies of Korean firefighters observed that Cd was significantly associated with an increased risk of MetS^23^ and hypertension.^22^ No significant associations were observed between blood Pb and Cd levels and the individual components of MetS. Experimental studies have shown that Pb and Cd exposure can affect lipid metabolism^52−55^ and potential mechanisms have been proposed to explain observed associations in human populations including the depletion of endogenous antioxidants, oxidative stress, and methylation of the genes involved in glucose and lipid metabolism.^55,56^

This study, unique in Czechia and Central Europe by the inclusion of 110 firefighters, presents concerning insights into their occupational exposure to hazardous metal(loid)s and their association with the serum lipid profile, indicating a risk factor for CVD. Moreover, this study is relevant also for other emergency service workers who can be exposed to fire emissions during rescue missions. However, it is important to note that single spot samples and cross-sectional design of the study might not mirror the firefighters’ real-life exposure during or immediately after fire suppression (which can be different from the levels observed in this study) and cannot confirm the causality of the observed relationships (although they are in line with experimental studies). Moreover, in August 2022, the first woman qualified for active fire response in Czechia, highlighting the need for future data collection of female firefighters.

Climate predictions go hand in hand with the growth of WUI and indicate a future increase in wildfire activity, leading to higher demands on fire rescue services^1^ and associated increased exposure of firefighters to harmful chemicals, including metal(loid)s. Our study found elevated urinary Pb levels in Czech firefighters compared to the control population, with firefighting activities showing a significant association with urinary Pb. Additionally, we observed associations between urinary levels of Pb and Cd, both individually and in complex mixtures with other metals and impairment of serum lipid profiles. The results suggest that firefighters, due to their occupational exposure, are more likely to develop unfavorable lipid profiles, increasing their risk of CVD in the future. Therefore, this study highlights the urgent need to mitigate such exposures and associated risks and proposes the following recommendations:

1) Identify dominant sources of exposure to metal(loid)s in firefighters’ environment (e.g., contaminated gear and surfaces in the vehicles and at the stations, dermal contact during decontamination procedures) and actively develop policies and procedures to minimize them.
2) Monitor internal levels of metal(loid)s, their biological effects as well as a number of firefighting incidents faced by individual firefighters to manage the occupational activities related to chronic exposures.
3) Provide information to firefighters on the risks of chronic exposure to metal(loid)s and their minimization through safety training, healthy lifestyle, and regular visits to a healthcare professional for preventive screenings.

## Data Availability

The data generated and analyzed during this study, including individual health, lifestyle, and chemical concentration data, are not publicly available due to sensitivity reasons. However, the data can be made available upon reasonable request to the corresponding author, subject to the establishment of data-sharing agreements. The data are securely stored in controlled access data storage at RECETOX, Brno, Czechia.

## Supporting Information Available

Details on sample collection and storage; questionnaire data (Table S1); details on quality control and quality assurance; formulas for correction of urine metal(loid)s for urine dilution (Table S2); directed acyclic graph (Figure S1); urinary levels of metal(loid)s adjusted for specific gravity (Table S3); urinary levels of metal(loid)s adjusted for creatinine (Table S4); Spearman correlation matrix (Figure S2); associations between population characteristics and urinary metal(loid)s (Table S5); associations between urinary metal(loid)s and serum lipids from regression models (Table S6); associations between urinary metal(loid)s and serum lipids from MLR and BWQS models (Table S7); BWQS mixture composition estimates (Table S8); BWQS results for metals–OH-PAH mixture (Table S9) (PDF)

## Notes

**Ethics approval.** The study was approved by the ELSPAC Ethics Committee, ethical approval number No: ELSPAC/EK/1/2019. All participants received an information brochure and participated in personal interviews in order to be fully informed about the study and their participation. In addition, informed consent was obtained from each participant before participation began. All data were pseudonymized to protect the identity of the participants.
