# Supplementary material for: Internal Flames: Metal(loid) Exposure Linked to Alteration of the Lipid Profile in Czech Male Firefighters (CELSPAC-FIREexpo Study)
Source: Environ Sci Technol Lett. 2024 Jun 12;11(7):679–86. doi: 10.1021/acs.estlett.4c00272 (PMC11238583; doi:10.1021/acs.estlett.4c00272)
Supplement: Supplementary file 1 — ez4c00272_si_001.pdf [file ez4c00272_si_001.pdf]

Supporting information

Internal Flames: Metal(loid) Exposure Linked to Alteration of Lipid Profile in Czech Male Firefighters (CELSPAC-FIREexpo Study)

Authors: Nina Pálešová[1], Katarína Řiháčková[1], Jan Kuta[1], Aleš Pindur[1,2,3], Ludmila Šebejová[1], Pavel Čupr[1]*

[1] RECETOX, Faculty of Science, Masaryk University, Kamenice 753/5, 625 00, Brno. Czech Republic

[2] Faculty of Sports Studies, Masaryk University, Kamenice 753/5, 625 00, Brno, Czech Republic

[3] Training Centre of Fire Rescue Service, Fire Rescue Service of the Czech Republic, Ministry of the Interior, Trnkova 85, 628 00, Brno, Czech Republic

(*) **corresponding author:** Pavel Čupr, RECETOX, Faculty of Science, Masaryk University, Kamenice 753/5, 624 00 Brno, Czech Republic, pavel.cupr@recetox.muni.cz

Journal: Environmental Science & Technology – Wildland Fires: Emissions, Chemistry, Contamination, Climate, and Human Health

Sample collection and storage

Recruitment occurred at the Training Center of the Fire Rescue Service in Brno (Czechia) for PROF and NEW FF, and at the Faculty of Sport, Masaryk University, Brno for CTRL.

Blood samples were collected by medical personnel in an operational ambulance. Urine samples were collected at the workplace by own urine collection following the instruction of medical personnel. In phases 1 and 3, morning void midstream urine was sampled, along with venous blood on an empty stomach. In phase 2, the sampling of morning void urine and venous blood on an empty stomach was not possible due to training schedule.

Venous blood for serum isolation was sampled in 7.5 mL S-Monovette® tube containing the Z-gel clotting activator. Each participant provided approximately 40 mL of midstream urine, which was collected in a 50 mL centrifuge tube. Both the venous blood and urine samples were immediately transported to laboratories in a cooling box set at 8°C.

Once the clot had formed in the venous blood tube, it was centrifuged at 2500×g and 20°C for 10 minutes. Subsequently, 0.5 mL aliquots were separated and placed into 1.2 mL cryotubes, which were then gradually frozen and stored in a biobank facility at -80°C for further analyses of the biomarkers and biochemical analysis. Similarly, the urine samples in 50 mL centrifuge tubes were divided into 1 mL aliquots in 1.2 mL cryotubes, frozen gradually, and stored in a biobank facility at -80°C until further analyses.

Table S1 – Results from the questionnaires [1].

		NEW FF	PROF	CTRL
**Participants that filled out questionnaires**		59	52	55
**Age (years)**	Median	24.5	28	26
	10th - 90th perc.	21 - 31	23 - 33	20 - 32
	Min. - Max.	19 - 34	20 - 35	18 - 35
**BMI (kg/m²)**	Median	26.3	26.2	24.6
	10th - 90th perc.	22.6 - 30.3	22.9 - 29	21.6 - 28.6
	Min. - Max.	20.7 - 33.4	21.1 - 32.2	18.4 - 30.9
**Health (subjective assessment, %)**	Always healthy and well	50.9	67.3	56.4
	Mostly healthy and well	49.2	32.7	41.8
	Often do not feel well	0	0	1.8
**Job (%)**	Firefighter	100	100	0
	Student	0	0	45.5
	IT	0	0	14.6
	Office	0	0	12.7
	Other	0	0	27.2
**Length of firefighting career (years)**	Median	0.5	3.3	0
	10th - 90th perc.	0.25 - 1	1 - 10	0
	Min. - Max.	0 - 5	0.5 - 14	0
**Smoking (%)**	Yes	0	0	0
	No	100	100	100
**Former smoking (%)**	Yes	11.9	19.2	7.3
	No	88.1	80.8	92.7
**If a former smoker (years since quitting)**	Median	4	0.5	3.5
	10th - 90th perc.	0.5 – 10	0.2 - 5	0.6 – 8
	Min. - Max	0.5 - 10	0.1 - 6	0.6 - 8
**Contact with a large fire in the last 6 months (%)**	Two or more times	22	59.6	5.5
	One time	32.2	21.2	0
	Never	45.8	19.2	90.9
**Contact with firefighting foams in the last year (%)**	Two or more times	0	34.6	0
	One time	25.4	40.4	1.8
	Never	69.5	25	98.2
**Diet (%)**	Mixed diet	100	100	100
	Vegetarian	0	0	0
	Vegan	0	0	0
**Use of food supplements (%)**	Yes	27.1	17.3	56.4
	No	72.9	82.7	43.6

Quality control and quality assurance

The concentration of three metals (Cd, Hg, and Pb) and one metalloid (As) in urine samples was determined by inductively coupled plasma mass spectrometry (Agilent 8900 ICP-MS/MS, Agilent Technologies) after 10x dilution of samples by solution containing deionized water, a nonionic surfactant Triton X-100 (0.04%), ammonia (1%), butanol (2%), EDTA (0.04%), and the appropriate internal standards for ICP-MS/MS (20 ng/mL of Sc, Ge, In, Lu and Bi). [2]

The samples underwent analysis at the Trace Analytical Laboratory at RECETOX MU (Brno, Czech Republic). This laboratory is accredited under ISO 17025.

Method was validated by analysis of samples spiked with known amount of analyte and by analysis of following certified reference materials (SERO AS, Norway): Seronorm™ Trace Elements Urine Level 1 and Seronorm™ Trace Elements Urine Level 2. Laboratory blanks (diluted aliquot of DI water) and certified reference materials (CRMs) were consistently analysed through the analytical sequence with frequency approximately one blank and one reference material for every 10 - 15 samples. The ICP-MS/MS measurements demonstrated recoveries typically ranging from 91 % to 112 % for both the spiked samples and the certified reference materials. Relative standard deviations calculated by repeatedly analysed CRMs during the measurement of the entire data set were within the range of single units of percent. Limits of detection for As, Cd, Hg and Pb were as follows: 0.03, 0.01, 0.01 and 0.02 ng/mL. Laboratory and method performances were successfully verified by participation in third-party proficiency testing, namely ICI-EQUAS under HBM4EU project and INSTAND EQUAS organized by German Medical Association.

The levels of total cholesterol (CHOL, mmol/L), low-density lipoprotein (LDL, mmol/L), high-density lipoprotein (HDL, mmol/L), and triglycerides (TG, mmol/L were measured spectrophotometrically with an Alinity c instrument (©Abbott, Illinois, U.S.A) in the private accredited laboratory, which conform the Quality Management system standard EN ISO 9001:2015.

Table S2 – Formulas used for correction of urine metal(loid)s for urine dilution [3].

Adjustment of metal(loid) levels for creatinine was performed by following formula:

$$c_{metal(loid)\_creat} = \frac{c_{metal(loid)}}{c_{creat}}$$

where $c_{metal(loid)\_creat}$ is creatinine adjusted metal(loid) urine concentration [$\mu g_{metal(loid)} / g_{creatinine}$], $c_{metal(loid)}$ is metal(loid) concentration in urine [ng/ml], and c_{creat} is urine creatinine concentration [g/l].

SG-corrected concentrations were calculated using following formula:

$$C_{SG} = \frac{C_i \times (SG_{ref} - 1)}{SG_{meas} - 1)}$$

where C_{SG} is the concentration standardized on SG, Ci is the measured concentration, SG_{meas} is the measured specific gravity, and SG_{ref} is the reference SG value. As a reference SG value, a mean SG value for all cohorts was used (1.02).

Figure S1 – Directed acyclic graph [4]

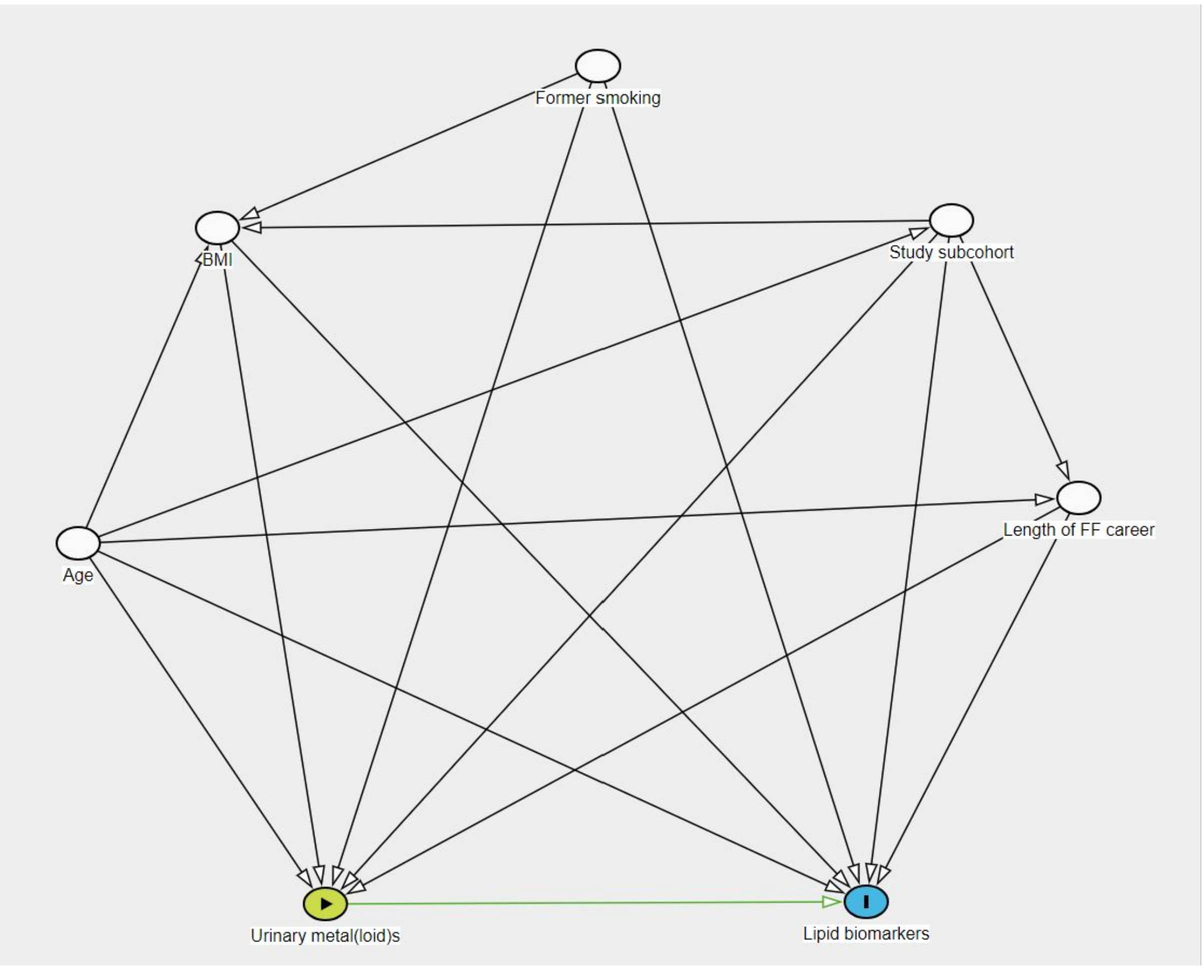

Table S3 – Urinary levels of metal(loid)s (ng/mL) adjusted for specific gravity in complete population ("All") and stratified for sub-cohorts ("CTRL" – control sub-cohort, "PROF" – sub-cohort of professional firefighters, "NEW FF" – sub-cohort of new firefighters in training)

**All (n=164)**	**DF**	**Mean**	**Sd**	**Min**	**Pctile[25]**	**Median**	**Pctile[75]**	**Max**
As_sg	100%	13	16	2.5	4.9	**6.3**	13	102
Cd_sg	100%	0.13	0.068	0.036	0.085	**0.11**	0.15	0.4

Hg_sg	100%	0.55	0.68	0.034	0.12	**0.29**	0.68	3.3
Pb_sg	100%	0.85	0.49	0.061	0.53	**0.74**	1.1	3.2
**CTRL (n=54)**								
As_sg	100%	15	19	2.6	5.5	**7.8**	14	102
Cd_sg	100%	0.13	0.071	0.036	0.083	**0.11**	0.17	0.38
Hg_sg	100%	0.44	0.47	0.034	0.13	**0.25**	0.54	2.5
Pb_sg	100%	0.69	0.52	0.21	0.38	**0.54**	0.78	3.2
**PROF (n=52)**								
As_sg	100%	14	18	2.7	5	**6.1**	11	70
Cd_sg	100%	0.14	0.075	0.044	0.089	**0.12**	0.18	0.4
Hg_sg	100%	0.66	0.86	0.039	0.12	**0.31**	0.74	3.3
Pb_sg	100%	0.86	0.39	0.061	0.59	**0.83**	1.1	2.2
**NEW FF (n=58)**								
As_sg	100%	11	11	2.5	4.2	**6**	12	52
Cd_sg	100%	0.12	0.057	0.036	0.085	**0.1**	0.14	0.33
Hg_sg	100%	0.55	0.66	0.038	0.12	**0.29**	0.72	2.9
Pb_sg	100%	1	0.5	0.26	0.66	**0.88**	1.2	2.7

Table S4 - Urinary levels of metal(loid)s (µg/g of creatinine) adjusted for creatinine in complete population ("All") and stratified for sub-cohorts ("CTRL" – control sub-cohort, "PROF" – sub-cohort of professional firefighters, "NEW FF" – sub-cohort of new firefighters in training)

**All (n=164)**	**DF**	**Mean**	**Sd**	**Min**	**Pctile[25]**	**Median**	**Pctile[75]**	**Max**
As_crea	100%	8.04	10.2	1.17	2.7	**4.27**	7.27	65.4
Cd_crea	100%	0.08	0.04	0.02	0.05	**0.07**	0.09	0.22
Hg_crea	100%	0.32	0.41	0.01	0.08	**0.19**	0.39	2.11
Pb_crea	100%	0.5	0.26	0.06	0.31	**0.44**	0.64	1.42
**CTRL (n=54)**								
As_crea	100%	8.18	11.1	1.37	2.67	**4.57**	7.54	65.4
Cd_crea	100%	0.07	0.03	0.03	0.05	**0.07**	0.08	0.16
Hg_crea	100%	0.23	0.21	0.01	0.08	**0.14**	0.35	1
Pb_crea	100%	0.35	0.21	0.09	0.25	**0.3**	0.38	1.26

**PROF (n=52)**								
As_crea	100%	9.58	12	1.76	3.17	**4.44**	8.46	46.6
Cd_crea	100%	0.09	0.04	0.03	0.07	**0.08**	0.11	0.22
Hg_crea	100%	0.42	0.52	0.03	0.08	**0.23**	0.46	2
Pb_crea	100%	0.57	0.26	0.06	0.4	**0.52**	0.71	1.42
**NEW (n=58)**								
As_crea	100%	6.53	6.85	1.17	2.47	**3.59**	6.51	33.7
Cd_crea	100%	0.07	0.03	0.02	0.05	**0.06**	0.08	0.19
Hg_crea	100%	0.33	0.41	0.02	0.07	**0.18**	0.39	2.11
Pb_crea	100%	0.58	0.26	0.17	0.39	**0.54**	0.7	1.38

Figure S2 – Spearman correlation matrix.

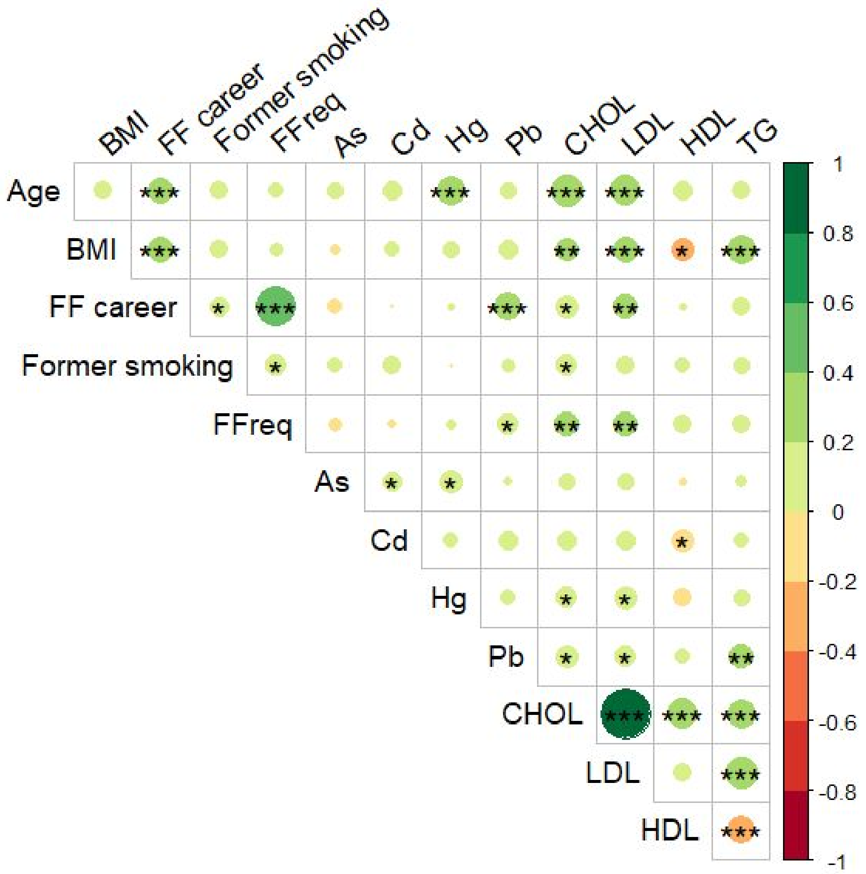

Table S5 – Associations between population characteristics and SG-adjusted urinary metal(loid)s. Expressed as relative change in urinary metal(loid)s per unit change in population characteristics. **Bold** refers to statistical significance ($p<0.05$)

Characteristics	As		Cd		Hg		Pb	
	β	p-value	β	p-value	β	p-value	β	p-value
**Age**	-5.47	0.487	**27.08**	**0.001**	**22.47**	**<0.001**	**31.71**	**<0.001**
**BMI**	**17.04**	**0.003**	-4.71	0.308	1.36	0.698	**-10.43**	**0.031**
**Sub-cohort**								
CTRL	reference		reference		reference		reference	
PROF	-6.42	0.571	9.89	0.397	2.85	0.760	**31.43**	**0.010**
NEW FF	-15.85	0.131	-10.45	0.309	5.45	0.514	**54.93**	**<0.001**
**Length of FF career**	-3.45	0.350	5.05	0.142	-0.18	0.940	**9.02**	**0.017**
**Contact with fire in the last 6 months**								
Never	reference		reference		reference		reference	
One time	-15.20	0.188	-6.29	0.588	2.85	0.760	**30.24**	**0.023**
Two or more times	-8.68	0.410	-0.23	0.982	5.45	0.514	13.57	0.214
**Former smoking**								
no	reference		reference		reference		reference	
yes	16.47	0.280	30.02	0.050	-0.60	0.953	13.79	0.328

Table S6 – Associations between urinary metal(loid)s ([ng/mL], categorized into quartiles) and serum lipids from regression models: β-coefficients and p-values. The model was adjusted for age, BMI, former smoking, length of firefighting career and sub-cohort (CTRL/PROF/NEW FF). Q1 – Q4: quartile 1 – quartile 4. **Bolt** refers to statistical significance ($p<0.05$). Results are expressed as percent change in lipid biomarker associated with quartile (Q2-Q4) compared to reference (Q1).

		CHOL		LDL		HDL		TG	
		β	p-value	β	p-value	β	p-value	β	p-value
**As**	**Q1**	*reference*							
	**Q2**	9.34	0.401	3.67	0.747	8.32	0.541	-2.26	0.853
	**Q3**	2.67	0.803	4.95	0.663	-6.34	0.609	-8.31	0.480
	**Q4**	**23.32**	**0.046**	21.85	0.073	-5.00	0.689	9.52	0.455
**Cd**	**Q1**	*reference*							
	**Q2**	-9.27	0.351	-9.49	0.361	-16.99	0.132	6.19	0.620
	**Q3**	8.18	0.458	8.12	0.482	-13.34	0.259	8.82	0.493
	**Q4**	3.01	0.782	7.37	0.527	**-29.90**	**0.006**	0.71	0.955
**Hg**	**Q1**	*reference*							
	**Q2**	-2.93	0.779	-8.41	0.427	4.15	0.75	-10.37	0.366

	**Q3**	5.56	0.616	10.03	0.394	-14.68	0.217	-4.42	0.713
	**Q4**	7.15	0.529	7.16	0.545	-19.22	0.111	15.28	0.258
**Pb**	**Q1**	*reference*							
	**Q2**	11.69	0.295	20.34	0.096	-0.82	0.949	14.72	0.261
	**Q3**	**30.97**	**0.013**	**33.15**	**0.012**	17.92	0.212	**31.19**	**0.030**
	**Q4**	**25.46**	**0.041**	23.85	0.066	11.07	0.444	**33.49**	**0.025**

Table S7 –Associations between urinary metal(loid)s (ng/mL) and serum lipids from MLR and BWQS models performed on reduced dataset (n=106, only CTRL and PROF): β-coefficients and 95% confidence intervals (CI, for MLR) and credibility intervals (CrI, for BWQS). The model was adjusted for age, BMI, former smoking, length of firefighting career and sub-cohort (CTRL/PROF). **Bolt** refers to statistical significance ($p<0.05$).

	**CHOL**	**LDL**	**HDL**	**TG**
	β (95% CI/CrI)	β (95% CI/CrI)	β (95% CI/CrI)	β (95% CI/CrI)
**As**	5.35 (-5.9 , 17.94)	5.14 (-6.14 , 17.77)	-7.31 (-18.69 , 5.66)	-0.9 (-12.45 , 12.17)
**Cd**	8.28 (-3.83 , 21.92)	8.9 (-3.32 , 22.67)	-10.72 (-21.97 , 2.17)	5.85 (-7.09 , 20.59)
**Hg**	3.16 (-12.23 , 21.25)	3.62 (-11.91 , 21.88)	**-20.18 (-33.48 , -4.22)**	**20.56 (1.42 , 43.31)**
**Pb**	**13.69 (0.83 , 28.2)**	10.42 (-2.23 , 24.71)	14.24 (-0.46 , 31.12)	**24.16 (9.33 , 40.99)**
**BWQS**	**14.7 (2.98 , 28.07)**	**20.34 (2.12 , 40.98)**	-10.85 (-25.07 , 10.44)	**15.28 (2.75 , 29.81)**

Table S8 –BWQS mixture composition estimates: weights (percentage rescaled between 0 to 1) for each component of metal(loid) mixture (M-M) and metal(loid)-OH-PAH mixture (M-PAH-M) in CHOL, LDL, HDL and TG. Intensity of the colour indicates increasing weight.

	**CHOL**		**LDL**		**HDL**		**TG**	
	M-M	M-PAH-M	M-M	M-PAH-M	M-M	M-PAH-M	M-M	M-PAH-M
As	0.307	0.116	0.258	0.136	0.163	0.106	0.173	0.095
Cd	0.187	0.091	0.207	0.110	0.421	0.127	0.152	0.094
Hg	0.188	0.101	0.214	0.109	0.321	0.117	0.248	0.127
Pb	0.319	0.145	0.322	0.111	0.096	0.099	0.427	0.155
1-OH-Naph	-	0.104	-	0.130	-	0.092	-	0.088
2-OH-Naph	-	0.121	-	0.133	-	0.094	-	0.101

1-OH-Pyr	-	0.090	-	0.063	-	0.096	-	0.083
2,3-OH-Phen	-	0.071	-	0.060	-	0.087	-	0.088
2-OH-Fluo	-	0.086	-	0.081	-	0.088	-	0.093
3-OH-Fluo	-	0.076	-	0.067	-	0.093	-	0.077

Table S9 – BWQS results for metals-OH-PAH mixture. **Bold** refers statistical significance (<0.05).

	**Mixture (Metal(loid)s + PAHs)**			
	**β**	**95% CrI**		
CHOL	**16.36**	**3.50**	**31.12**	*
LDL	**18.81**	**4.56**	**35.73**	*
HDL	-2.31	-18.81	14.08	
TG	10.97	-2.47	27.81	

References

(1) Řiháčková, K.; Pindur, A.; Komprdová, K.; Pálešová, N.; Kohoutek, J.; Šenk, P.; Navrátilová, J.; Andrýsková, L.; Šebejová, L.; Hůlek, R.; Ismael, M.; Čupr, P. The Exposure of Czech Firefighters to Perfluoroalkyl Substances and Polycyclic Aromatic Hydrocarbons: CELSPAC – FIREexpo Case-Control Human Biomonitoring Study. **2023**, *881* (April). https://doi.org/10.1016/j.scitotenv.2023.163298.

(2) Válková, L.; Emmer, J.; Kuta, J.; Goldbergová, M. P. Determination of Metal Ion Levels in Circulation in Patients with Joint Replacement. *Hem. Ind.* **2024**, *78* (1S), 45.

(3) Sauvé, J. F.; Lévesque, M.; Huard, M.; Drolet, D.; Lavoué, J.; Tardif, R.; Truchon, G. Creatinine and Specific Gravity Normalization in Biological Monitoring of Occupational Exposures. *J. Occup. Environ. Hyg.* **2015**, *12* (2), 123–129. https://doi.org/10.1080/15459624.2014.955179.

(4) Shrier, I.; Platt, R. W. Reducing Bias through Directed Acyclic Graphs. *BMC Med. Res. Methodol.* **2008**, *8*, 1–15. https://doi.org/10.1186/1471-2288-8-70.
